# Modulating pro-adhesive nature of metallic surfaces through a polypeptide coupling via diazonium chemistry

**DOI:** 10.1038/s41598-023-45694-z

**Published:** 2023-10-26

**Authors:** Taral Patel, Magdalena Skonieczna, Roman Turczyn, Katarzyna Krukiewicz

**Affiliations:** 1 Department of Physical Chemistry and Technology of Polymers, Silesian University of Technology, M. Strzody 9, 44-100 Gliwice, Poland; 2 Joint Doctoral School, Silesian University of Technology, Akademicka 2A, 44-100 Gliwice, Poland; 3 Biotechnology Centre, Silesian University of Technology, Krzywoustego 8, 44-100 Gliwice, Poland; 4 Department of Systems Biology and Engineering, Silesian University of Technology, Akademicka 16, 44-100 Gliwice, Poland; 5 Centre for Organic and Nanohybrid Electronics, Silesian University of Technology, Konarskiego 22B, 44-100 Gliwice, Poland

**Keywords:** Implants, Electrochemistry

## Abstract

The design of biomaterials able to facilitate cell adhesion is critical in the field of tissue engineering. Precise control of surface chemistry at the material/tissue interface plays a major role in enhancing the interactions between a biomaterial and living cells. Bio-integration is particularly important in case of various electrotherapies, since a close contact between tissue and electrode's surface facilitates treatment. A promising approach towards surface biofunctionalization involves the electrografting of diazonium salts followed by the modification of organic layer with pro-adhesive polypeptides. This study focuses on the modification of platinum electrodes with a 4-nitrobenzenediazonium layer, which is then converted to the aminobenzene moiety. The electrodes are further biofunctionalized with polypeptides (polylysine and polylysine/laminin) to enhance cell adhesion. This study also explores the differences between physical and chemical coupling of selected polypeptides to modulate pro-adhesive nature of Pt electrodes with respect to human neuroblastoma SH-SY5Y cells and U87 astrocytes. Our results demonstrate the significant enhancement in cell adhesion for biofunctionalized electrodes, with more amplified adhesion noted for covalently coupled polypeptides. The implications of this research are crucial for the development of more effective and functional biomaterials, particularly biomedical electrodes, which have the potential to advance the field of bioelectronics and improve patients' outcomes.

## Introduction

Since proper cell adhesion is vital to assure tissue regeneration and growth, the affinity of the cell to bind to a substrate is a key point in the design of biomaterials^1^. Therefore, materials employed in the design of scaffolds intended for tissue regeneration are expected to act as substrates promoting the adhesion and proliferation of cells^2^. Firm adhesion of cells is particularly important in case of various electrotherapies, where electrical energy is used as a medical treatment. Electrical stimulation has been recently introduced as an effective tool to regulate cell behavior in tissue engineering, showing its efficiency in wound healing, treatment of neurological disorders, and bone regeneration, among others^3–5^. Since a close contact between tissue and an electrode's surface is essential to provide an efficient treatment, recent research studies focus on developing electrode coating materials able to promote cellular adhesion to the surface of the electrodes^6^.

The tendency of a cell to adhere to a surface depends on many factors, including physical properties of the surface (roughness, hardness, elasticity, wettability), its chemical structure (surface energy, surface charge, functional groups), or biological affinity (presence of biomolecules that are able to specifically interact with a cell membrane)^7^. Consequently, the most common approaches used to increase cell adhesion involve increasing surface roughness and elasticity, modulating wettability, introducing positively charged functional groups, as well as immobilizing pro-adhesive biomolecules^8^. One of common methods is to use the components of extracellular matrix (ECM), particularly proteins and peptides, as pro-adhesive coatings^9^. Poly-L-lysine (PLL) has a widespread use in assisting cell adhesion and proliferation, and two major reasons of employing PLL for cell cultivation are: (1) favorable interactions between positively charged amino groups in PLL and negatively charged cell surfaces and (2) economic viability^7^. Another ECM glycoprotein promoting cell adhesion is laminin, which is a high molecular mass protein with rod-like chain domains promoting cell binding through its interactions with transmembrane receptors (integrins)^10^.

Immobilization of proteins on the substrate using conventional methods, like non-covalent adsorption, deposition, and physical entrapment, is based on weak interactions, hence the stability of as-formed bioactive layer is poor^11^. Therefore, covalent immobilizations can be an efficient route to bind proteins to the material creating a peptide-modified surface that improves adhesion of cells. Some researchers have already reported that covalent immobilization of laminin on the surface of silicon provides beneficial in vitro and in vivo activity, particularly for neural progenitor cells^12^. Proteins having lysine residues in the exterior are used as anchoring points for agents like ethyl(dimethylaminopropyl) carbodiimide (EDC) and N-hydroxysuccinimide (NHS). Consequently, EDC/NHS chemistry is applicable for the immobilization of laminin as well as polylysine^13^. Proteins need to be dissolved in an appropriate buffer solution and put in contact with EDC and NHS to form a strong amide bond allowing firm attachment to the surface^14^. Surface modification using diazonium salts is a cornerstone in the field of materials science, offering a powerful tool for tailoring the surface properties of diverse substrates^15^. This methodology relies on the chemical reactivity of diazonium salts, which are organic compounds containing a diazo group (N_2_^+^), able to be covalently attached to substrates such as metals, semiconductors, and carbon-based materials^16^. The versatility of diazonium salt-based surface modification lies in its ability to precisely control and engineer a wide array of surface properties, including but not limited to hydrophilicity, roughness, chemical reactivity, and biocompatibility, through the judicious selection of diazonium compounds with appropriate functional groups^17^. One of the advantages of this technique is the formation of a covalent bond between an organic compound and a substrate, ensuring exceptional adhesion and long-term stability of modified surfaces^18^. Several techniques can be employed for diazonium salt-based surface modification, including immersion methods, vapor phase modification, and solution-based approaches^19^. However, electrochemical deposition stands out as a superior method due to its control over the modification process, leading to the formation of uniform layers with controlled thickness. Additionally, it is compatible with a wide range of electrically conducting substrates, making it a versatile choice for various applications^20–22^. Electrochemical deposition often involves milder reaction conditions and produces fewer environmentally harmful byproducts, aligning with green chemistry principles^17^.

In our work, we hypothesized that by the modification of the surface of Pt electrodes with an organic layer formed from electrografted 4-nitrobenzene tetrafluoroborate, electrochemically converted into 4-aminobenzene moiety, we would be able to covalently immobilize pro-adhesive polypeptides (polylysine and laminin) to provide stable electrode coating exhibiting pro-adherent properties towards mammalian cells. The aim of our work was to improve the design of neural electrodes, hence human neuroblastoma cell line SH-SY5Y and U87 astrocytes were employed as model cell lines to investigate cell adhesion properties of biofunctionalized coatings. Although neural cells are the usual target for electrical stimulation, astrocytes outnumber neurons in the human brain and support their activity through affecting neurotransmission, cell signaling, metabolite and electrolyte homeostasis, inflammation, and synapse modulation^23^. Therefore, the biological characterization of any biomaterial intended for the use within the central nervous system should include not only excitable neural cells, but also co-existing glial cell, particularly astrocytes. Here, we focused on the effect of polypeptides covalently attached to platinum electrode surfaces in relation to cell adhesion and cell morphology, which is related to its motility and migration potential.

## Materials and methods

### Reagents and solutions

4-nitrobenzenediazonium tetrafluoroborate (NBD, 97%) and tetrabutylammonium tetrafluoroborate (Bu_4_NBF_4_, ≥ 99%) were acquired from Sigma Aldrich (Missouri, USA). Bu_4_NBF_4_ was vacuum dried before use. Phosphate buffer saline (PBS) tablets were obtained from Fisher Scientific, one tablet provided a buffer solution containing 0.01 M phosphate buffer, 0.0027 M potassium chloride, 0.137 M sodium chloride when it was dissolved in 200 ml ultrapure water. Potassium chloride (KCl), N-ethyl-N′-(3-dimethylaminopropyl)carbodiimide HCl (EDC, 98% pure) were acquired from Acros Organics. Poly-L-lysine (PL) (0.1% w/v) and poly-D-lysine & laminin (PL/LM) solution were obtained from Sigma-Aldrich. Ultrapure water, acetonitrile (ACN, 99.9%, Sigma-Aldrich) and absolute ethanol (EtOH, Sigma-Aldrich) were utilized as the solvents. All the reagents were used as obtained.

### Electrochemical studies

Electrochemical reduction of 4-nitrobenzenediazonium tetrafluoroborate (NBD) was performed with the use of a potentiostat (CHI 660C potentiostat, Texas, USA). A three-electrode electrochemical cell consisting of Ag wire as a pseudoreference electrode, platinum disk as an auxiliary electrode (1 cm × 1 cm, Mennica Polska, Poland), along with thermanox coverslips (NUNC Brand products Rochester, NY, USA) coated with platinum thorough a sputter-coating process (Q150R rotary pump, Quorum technologies, UK) as a working electrode were used for the measurements. The potential of the pseudoreference electrode was monitored with the use of the ferrocene. Cyclic voltammograms were obtained by a potential sweep scanning from −0.9 to 0.5 V (vs. Ag) in the solution containing 3 mM NBD dissolved in 0.1 M Bu_4_NBF_4_/ACN. Cyclic voltammetric scanning was performed until 5 cycles at the scan rate of 0.05 V/s. The conversion of the 4-nitrobenzene (NB) group into the 4-aminobenzene (AB) group was performed using the aforementioned three-electrode configuration. The reaction took place in a solution consisting of 0.1 M KCl aqueous solution mixed with ethanol in a 9:1 volume ratio. Cyclic voltammograms were obtained by a potential sweep from 0.2 to −1.0 V (vs. Ag) for 2 cycles at the scan rate of 0.05 V/s.

### Polypeptides biofunctionalization

Poly-L-lysine (PL) at a concentration of 0.1% w/v and a combination of Poly-D-lysine and laminin (PL/LM) were used as coatings for the NB-modified platinum electrodes. Two methods were employed for the coating process: drop-casting and EDC coupling. In the drop-casting method, 40 µL of either PL or PL/LM solution was used to coat the modified electrodes. After one hour, the electrodes were subjected to three washes with ultrapure water and were left to dry in air. In the EDC coupling method, the electrodes were submerged into a solution containing 6 mM EDC in PBS and either PL or PL/LM solution. After a 20 min immersion period, the electrodes were rinsed with ultrapure water with further air-drying at 20 °C.

### Characterization

The optical profilometer Profilm 3D (Filmetrics), equipped with a phase shift interferometry (PSI) mode, was used for morphological characterization. ISO 25,178 standards were followed to obtain the surface roughness (S_a_). To analyze the spectrochemical properties of coatings, FTIR ATR spectroscopy was conducted using an IR Perkin Elmer spectrum two spectrometer over a range of 650–4000 cm^−1^, and Renishaw inVia instrument equipped with 514 nm excitation laser was utilized to attain Raman spectra, in range of 400–1600 cm^−1^. Contact angle (θ) measurements were conducted at 20 °C via an optical goniometer (OCA15 Dataphysisc) using ultrapure water. Electrochemical impedance (EIS) spectra were collected by means of a CHI 660c potentiostat in PBS solution within the frequency range from 10 kHz to 0.1 Hz, with an AC amplitude of 50 mV. Data fitting was performed using EIS spectrum analyzer software with NM Simp algorithm^24^. The experimental data were fitted in the circuit consisting of solution resistance (R_s_), charge transfer resistance (R_CT_), and two constant phase elements (CPE_1_, CPE_2_), this circuit is a modified version of the Randles circuit describing the capacitance referring to the double layer capacitors^25^. The experiments were performed at normal temperature and pressure, as defined by the National Institute of Standards and Technology (20 °C, 1 atm) and without any purging prior to the measurement.

### Cell culture studies

Biological characterization involved the utilization of commercially available human cell lines, namely SH-SY5Y (CRL-2266, ATCC) derived from neuroblastoma and U87 (HBA-14, ATCC) derived from glioblastoma. Both cell lines were purchased from Sigma Aldrich. SH-SY5Y cells were cultured in Dulbecco's Modified Eagle Medium/Nutrient Mixture F-12 (DMEM/F12, Sigma-Aldrich), while U87 cells were grown in Minimum Essential Medium Eagle (EMEM, Sigma Aldrich). Both media were augmented with 10% fetal bovine serum (Gibco, Thermo Fisher Scientific) along with 1% penicillin/streptomycin solution (PS, Sigma-Aldrich). Cell cultures were maintained at 37 °C, 80% humid atmosphere with 5% CO_2_ concentration using a Heracell™ 150i incubator (Thermo Fisher Scientific).

In the experimental phase, cells were subjected to trypsinization through a 0.25% trypsin–EDTA solution (Sigma-Aldrich) and the enzymatic reaction was stopped by adding twice amount of culture medium. Then, 5 × 10^4^ cells were evenly distributed in a 100 µl drop on 1 cm × 1 cm samples in 12-well plates (Biologix) and allowed to culture for 1 h. Afterward, the wells were thoroughly washed with 1 × Dulbecco's phosphate-buffered saline (DPBS, PAN Biotech) and were sustained in DPBS solution for the imaging. For cell counting, 10 images per sample were collected and compared to the plastic control. Automatic cell counting and analysis of confluence were conducted using a Live Cell Analyzer (JuLI™ FL, NanoEnTek) and ImageJ (NIH).

## Results and discussion

### Surface modification

**Figure 1:**
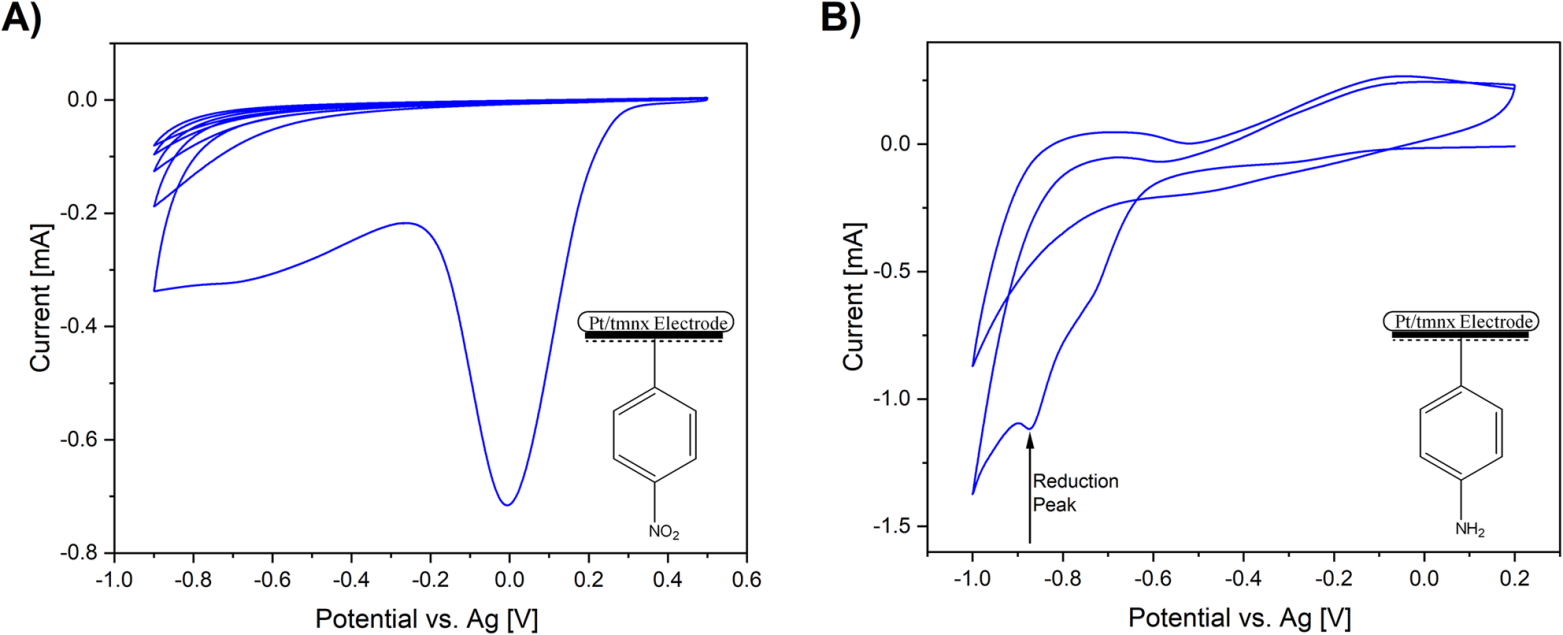
Cyclic voltammogram showing the reduction of 3 mM NBD in 0.1 M Bu_4_NBF_4_/ACN at the scan rate of 50 mV/s (**A**). Cyclic voltammogram of electrochemical reduction of NB to AB in 0.1 M KCl/water:ethanol (9:1) at the scan rate of 50 mV/s (**B**).

Electrochemical deposition of NBD was carried out on Pt-coated Thermanox coverslips (Pt/tmnx) treated for cell culture with the use of cyclic voltammetry (CV). It is well-known that the reduction of aryldiazonium salts involves a single-electron transfer from the cathode to the aryldiazonium salt, releasing a nitrogen molecule and forming an aryl radical, followed by the formation of a covalent bond between the electrode surface and the aryl group^26^. As shown in Fig. 1A, the reduction of NBD is prominent in the first cycle at 0 V (vs. Ag), as at this potential a broad irreversible peak appears indicating the reduction and covalent attachment of NB to Pt/tmnx surface. The rapid nature of reduction of NBD can be confirmed by the disappearances of the reduction peak in the subsequent CV cycles.

Electrografted NB groups were further reduced to AB groups using 0.1 M KCl/water:ethanol (9:1) solution (Fig. 1B) The reduction peak at −0.9 V (vs. Ag) in the first cycle states that the reduction process takes place on the surface of the electrode. The process of reducing NB involves two steps: first, a four-electron electron exchange process is used to reduce NB to 4-phenylhydroxylamine (PHA) with the formation of an intermediate called 4-nitrosobenzene (NSB). PHA then undergoes a two-electron reduction process to become AB. Due to a fast conversion of NSB to unstable PHA, which is further converted into AB, neither NSB nor PHA can be identified^27^. Both steps of reduction had an impact on the electrical properties of the electrode, as evidenced by the change in a charge transfer resistance and capacitance as evaluated by the EIS (Figure S1, Table S2). Interestingly, the modification of Pt with both NB and AB leads to the decrease in the impedance module at the low frequency range (< 10 Hz), which is relevant in the case of a low frequency transcutaneous electrical nerve stimulation applicable in the treatment of acute and chronic pain^28^.

### Biofunctionalization of surface

**Figure 2:**
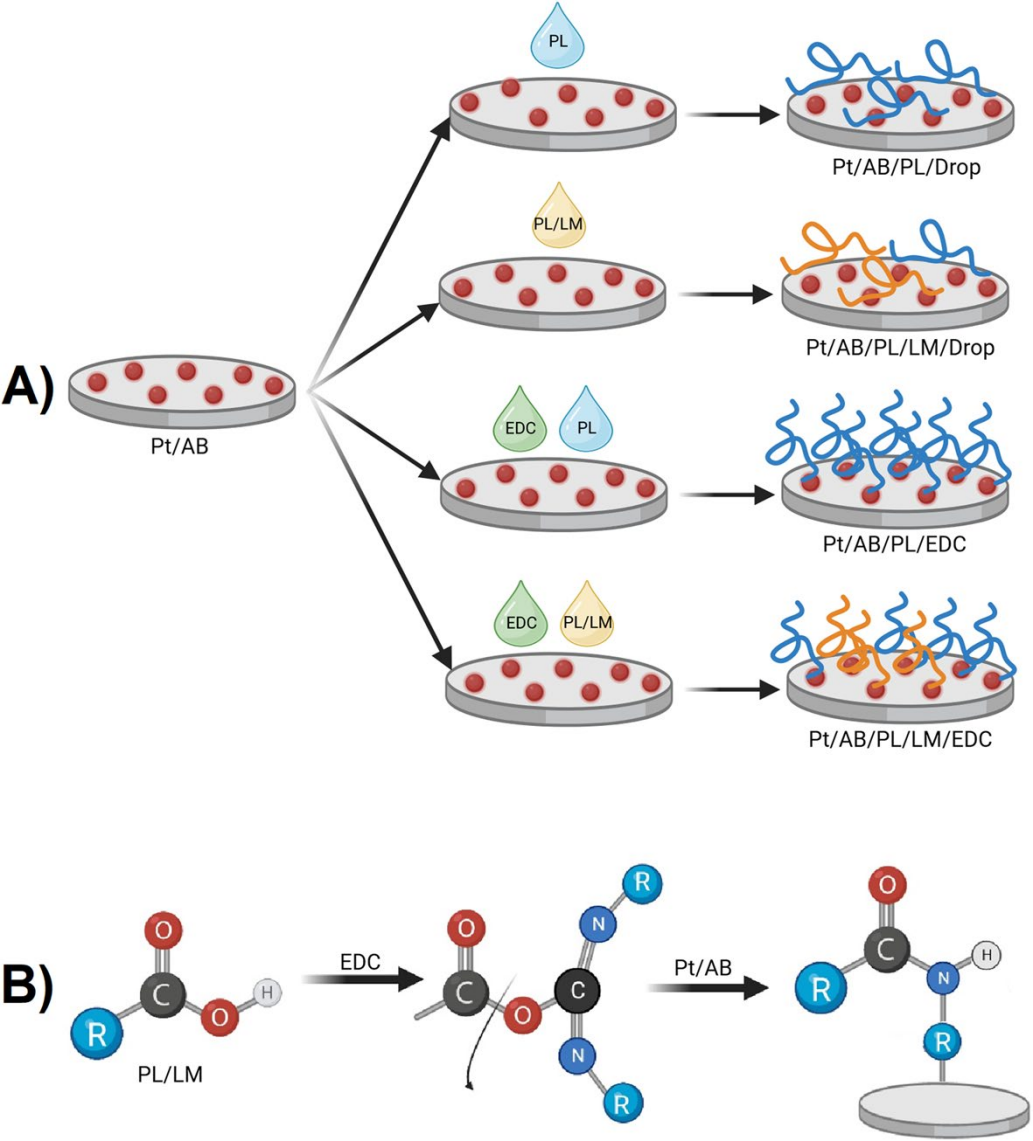
Schematic representation of the process of biofunctionalization of AB-modified Pt electrodes with either PL or PL/LM using either drop casting or EDC coupling methods (**A**). Schematic representation of the mechanism of EDC coupling (**B**).

To enhance the adhesive properties of the electrodes, two polypeptides known to increase cell attachment of the surface were used to modify the electrodes, namely polylysine (PL) and polylysine/laminin (PL/LM). A drop casting method as well as a covalent coupling method were used to coat the electrodes (Fig. 2). Drop casting is a well-known technique suitable for the deposition of a variety of compounds onto different substrates. In this method, consecutive drops of a given solution are placed onto surface forming a thin layer after evaporation of the solvent. Drop casting is usually used to coat different substrates as a simple and rapid application, however, it has a disadvantage of forming non-uniform layers with not well controlled thickness^29^. Considering more uniform and stable attachment of polypeptides onto the surface, we investigated EDC coupling mechanism that involves the use of a crosslinker to combine polypeptides with the amino groups present on the surface^13^. EDC reacts with the carboxylic acid group present in both polylysine and laminin to create a favorable site for binding for amino groups present on the surface of the electrode modified with AB forming an O-acylisourea intermediate^30^. The amino group attaches to the site and EDC is removed as a byproduct. Due to the formation of covalent bonds between functionalized electrodes and polypeptides, a stable coating is expected to be formed. Even though coating the surface of a Pt electrode with a polypeptide layer increases its charge transfer resistance (Table S1), the electrical parameters of AB/Pl and AB/PL/LM do not compromise its applicability in electrical stimulation, particularly in the high frequency region (> 1000 Hz), which could be used for analgesic modality^31^.

### Spectroscopic characterization

**Figure 3:**
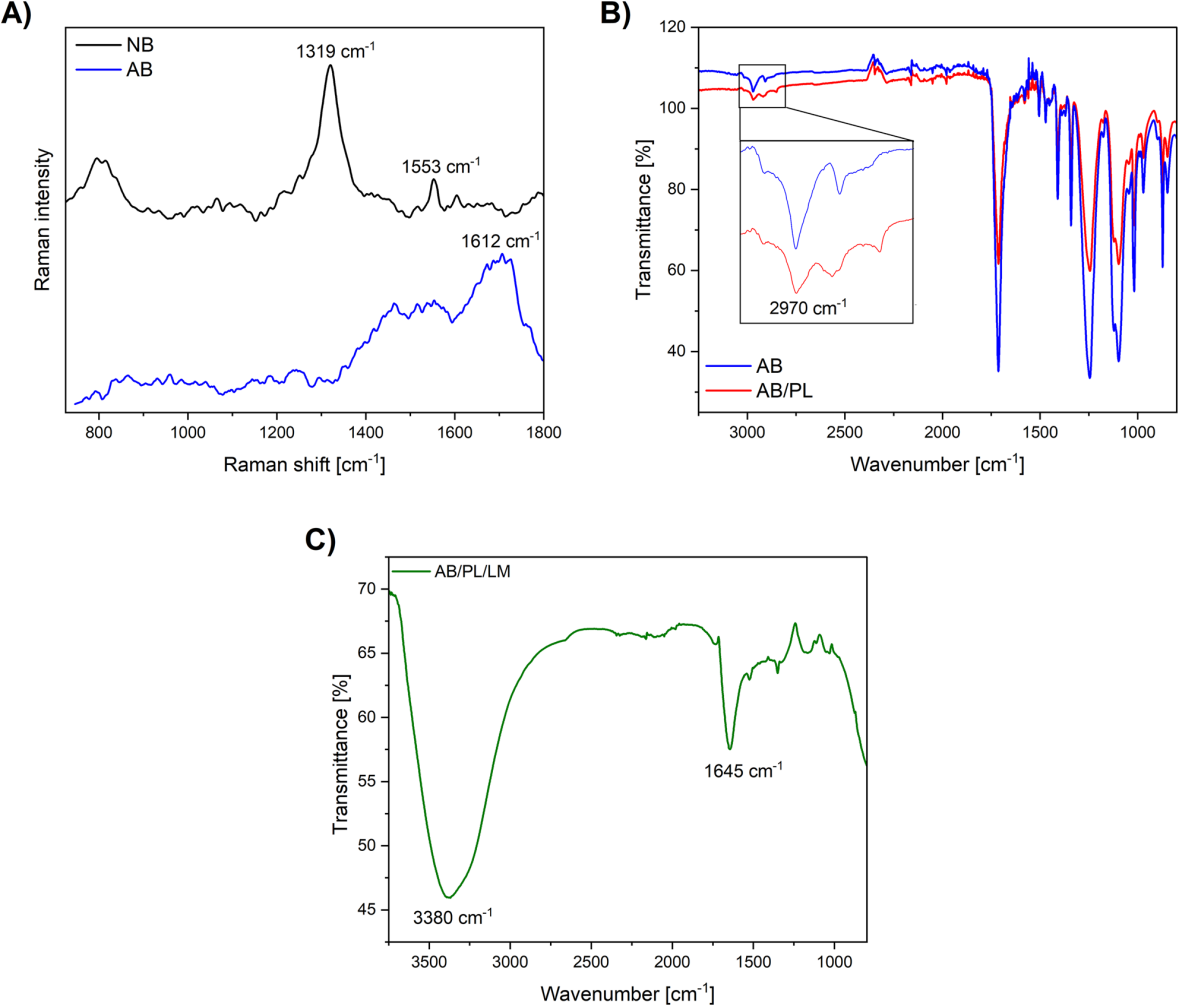
Raman spectrum of NB and AB (**A**), FTIR spectra of AB/PL with the insight comparing FTIR peak of C–H in AB and AB/PL (**B**), FTIR spectrum of AB/PL/LM with marked characteristic peaks (**C**).

The presence of NB and AB groups on the surface of Pt/tmnx was investigated via Raman and Fourier-transform infrared spectroscopy (FTIR). Figure 3A shows the Raman spectra of NB and AB modified surface, respectively, with marked peaks at 1319 cm^−1^ (C-NO_2_, sym.), 1553 cm^−1^ (C-NO_2_, asym.) for NB and 1612 cm^−1^ (C-NH deformations) for AB. The presence of polypeptides on the electrografted electrode surface was investigated via FTIR spectroscopy. Since in the first step PL was applied on the surface of AB-modified Pt electrode, its FTIR spectrum (Fig. 3B) exhibits AB-derived signals, but with a lower intensity, namely asymmetric C–H stretching vibration at 2970 cm^−1^, and C-NH stretching at 1710 cm^−1^. These signals overlay with the signals characteristic for PL, particularly amide I absorption frequency correlated with the presence of β-sheets (1620 cm^−1^ and 1680 cm^−1^)^32^. The spectra of PL and AB could be distinguished by the analysis of CH_2_ stretching modes in the range 3050 cm^−1^–2800 cm^−1^ (Fig. 3B inset)^32^, which are more complex and more pronounced in PL. Figure 3C shows the attachment of PL/LM onto AB surface with confirmed by the presence of characteristic peaks at 3380 cm^−1^ (N–H stretching), 1645 cm^−1^ (C=O stretching of amide in laminin).

### Surface characterization

**Figure 4:**
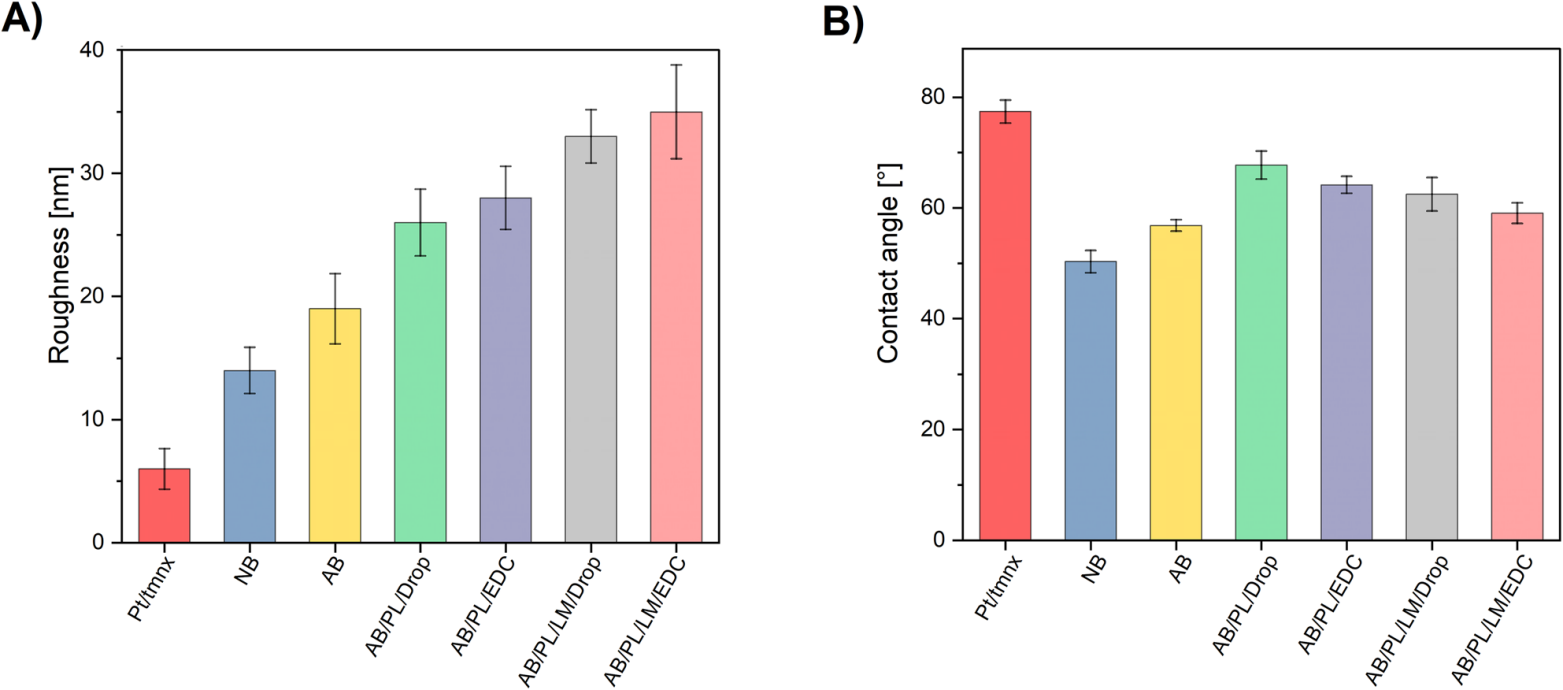
Surface roughness (**A**) and contact angle (**B**) comparison between Pt/tmnx and functionalized electrodes.

Figure 4A shows the comparison of surface roughness between bare Pt/tmnx and modified electrodes. The surface of NB and AB shows increased roughness when compared with unmodified Pt/tmnx (6.0 ± 1.7 nm), having S_a_ of 14.0 ± 1.9 nm (NB), and 19.1 ± 2.9 nm (AB), respectively. After biofunctionalization of electrodes with adhesive polypeptides, a significant increase in surface roughness is observed, with AB/PL/LM deposited through a drop casting showing almost a twofold increase in roughness (33.4 ± 2.2 nm) when compared to AB (19.1 ± 2.9 nm). This study also supports the initial claim on the increased roughness of polylysine and laminin coatings deposited by a coupling method with S_a_ of 28.1 ± 2.6 nm and 35.3 ± 3.8 nm for AB/PL/EDC and AB/PL/LM/EDC, respectively. Roughness plays an important role in determining the adhesive nature of any surface^33^. In a study on the adhesion of rat cortical neurons on silica wafers, several substrates with different roughness (from 10 nm to 250 nm) were examined, revealing the optimum surface roughness range between 20 nm and 100 nm that promotes cell adhesion^34^. The results of another study indicated surface roughness of 20–50 nm as the optimal for the adhesion of neural cells^35^. Nanotopography was also found to affect the adhesion of astrocytes, which was promoted on the surfaces modified with an array of nano pores (20 nm diameter), exhibiting the roughness of approx. 5 nm^36^. Even though these values are much lower than the size of the cells, it is expected that a key role in cell adhesion is played by nanosized cellular structure called filopodia, which are able to interact with the surface features as small as 10 nm in height. Because surface roughness is not the only parameter influencing the neural cell adhesion, in the next step of our research, surface wettability was assessed.

Figure 4B shows the comparison of contact angles of bare Pt/tmnx electrode and functionalized electrodes. Contact angle of a bare Pt/tmnx electrode is 77° ± 2°, which is high comparing with the electrodes after modification with NB (50° ± 2°) and its reduction to AB (60° ± 1°). Further biofunctionalization of samples with PL and PL/LM resulted in a slight increase in θ (67° ± 1° for AB/PL/Drop and 62° ± 2° for AB/PL/LM/Drop, as well as 64° ± 1° for AB/PL/EDC and 59° ± 2° for AB/PL/LM/EDC), but still below the contact angle of unmodified Pt electrode. Researchers reviled that extreme hydrophilic (θ < 2°) and extreme hydrophobic surfaces (θ > 150°) are not favorable for cell adhesion^7^. Another study indicated the water contact angle of 55° as the optimum value to facilitate adhesion of neural cells^37^. Since for both “Drop” and “EDC” layers of AB/PL and AB/PL/LM contact angle decreased by 10° when compared with a bare Pt electrode, oscillating around the value of 60°, these changes are anticipated to provide a balanced hydrophilic surface which is optimal for the cell adhesion and growth^38^.

### Cell adhesion

**Figure 5:**
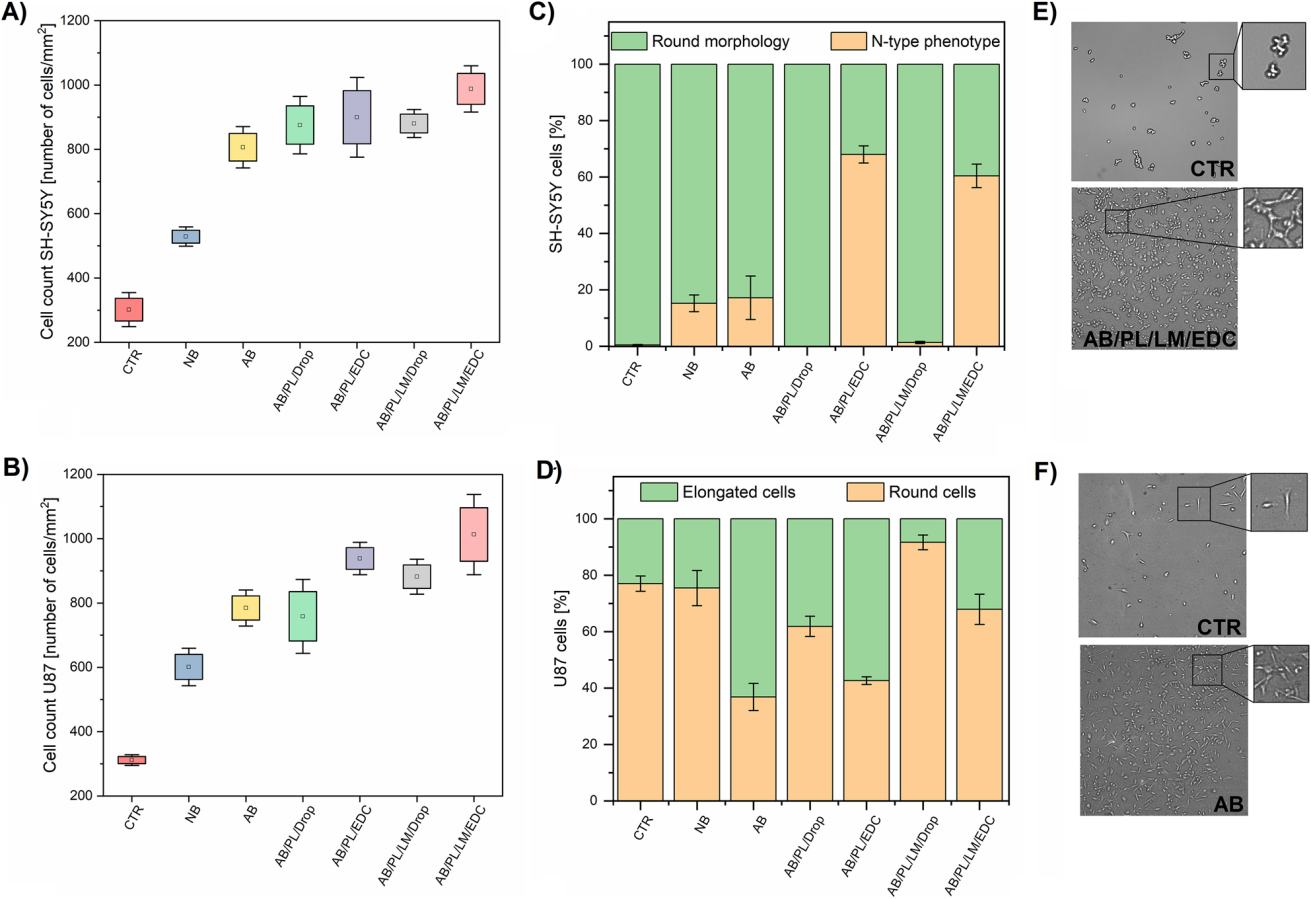
Cell density of SH-SY5Y (**A**) and U87 cells (**B**) as number of cells per mm^2^ for modified electrodes, compared to a tissue culture plastic control sample (CTR); a box represents a standard error with a mean value as a square in the center of the box. Percentage of SH-SY5Y cells with a round and N-type phenotype (**C**). Percentage of U87 cells with elongate or round morphology (**D**). Representative optical images of SH-SY5Y cells cultured on a control sample and an electrode modified with AB/PL/LM/EDC (**E**). Representative optical images of U87 cells cultured on a control sample and an electrode modified with AB (**F**).

The effect of cell adhesion after surface biofunctionalization was investigated with respect to human neuroblastoma cells SH-SY5Y and human glioblastoma cells U87 (Fig. 5). The quantity of attached cells on NB and AB samples (550 cells/mm^2^ for NB and 750 cells/mm^2^ for AB, respectively) is significantly higher than in the case of a tissue culture plastic control (300 cells/mm^2^). For the samples biofunctionalized with PL via a drop casting method (AB/PL/Drop), SH-SY5Y and U87 cell count is around 800 cells/mm^2^. Similar results were observed using AB/PL/LM/Drop with the SH-SY5Y and U87 cell count about 850 cells/mm^2^, whereas the covalently coupled polypeptide samples showed higher cell adhesion, as for both PL and PL/LM modified samples the SH-SY5Y and U87 cell counts were close to 1000 cells/mm^2^.

Microscopic investigation of SH-SY5Y and U87 cells attachment to the surface revealed the difference between drop casting and EDC covalent coupling attachment protocols for pro-adhesive polypeptides. For instance, the SH-SY5Y and U87 cell counts for samples AB/PL/Drop and AB/PL/LM/Drop were noted with over threefold increase with respect to the control. Simultaneously, for the covalently coupled samples (AB/PL/EDC and AB/PL/LM/EDC) the cell count significantly increased when compared with the control. Furthermore, the coupling of PL/LM to the surface resulted in the maximum number of adhered cells (1100 cells/mm^2^) when compared to all polypeptide-modified samples, manifesting pro-adhesive nature and covalent coupling ability of polypeptides. A handful of explored EDC coupling protocols for polypeptides adhesion revealed the superiority of amide coupling via EDC chemistry^12,39,40^. In our studies for SH-SY5Y cells, the adhesive nature of the biofunctionalized electrodes surged after covalent coupling of PL and PL/LM. Similar outcomes persisted with U87 cells, which was obvious when comparing the number of cells for AB/PL/EDC and AB/PL/LM/EDC samples. Consequently, aforementioned effect of polypeptides enhancing the neuronal cell adhesion is evident here, particularly for the electrodes coated with PL/LM^41^.

Apart from the number of cells attached to the surface, optical images provided information about morphology of cells and their differentiation (Fig. 5C–F). For instance, SH-SY5Y cells cultured on the surface of bare Pt are characterized by a round shape without the presence of any cell processes, namely neurites, which could indicate their weak adhesion to the substrate. On the other hand, SH-SY5Y cells cultured on the surface of Pt electrode modified with PL/LM layer depicted the neuron-type (N-type) phenotype, characteristic for neuroblasts having the ability to differentiate into neuron-like cells^42^. Even though the population of SH-SY5Y cells is generally not uniform, and it is expected to find different types of cell morphologies on the surface of a single sample, N-type phenotype was more prevalent on the surfaces modified with polypeptides via EDC chemistry, achieving 60% of total cell population, than on the surfaces modified through a drop-casting method (0–1.5%). Since EDC is able to couple peptides from a definite position (C-terminus), it is expected that the peptide coating formed via EDC chemistry will be arranged on the surface in the form of brush-like structures, which could provide an additional dimension to facilitate cell attachment, growth, and differentiation^43^.

A similar observation was also made in the case of U87 cells, which were found to express either round or elongated morphology. It is known that U87 cells with rounded shape are more prone to form large and dense cell clusters, while the elongation of U87 cells (migratory phenotype) is associated with their higher motility and migration potential^44^. Among experimental samples, it was AB/PL/LM/Drop that suppressed the migration potential of U87 cells (the lowest percentage of elongated cells, namely 8%), most probably due to a strong adhesion between LM and U87. On the other hand, U87 cells present on the surface of AB sample were observed to exist mainly in an elongated form (63%), which was supposed to facilitate their motility. High motility of glial cells is the reason of the malignancy of the brain cancer (glioblastoma), therefore, the possibility of functionalization of the surface of a material to suppress the migration of glial cells could be particularly important in electrochemotherapy to prevent metastases^45^.

**Figure 6:**
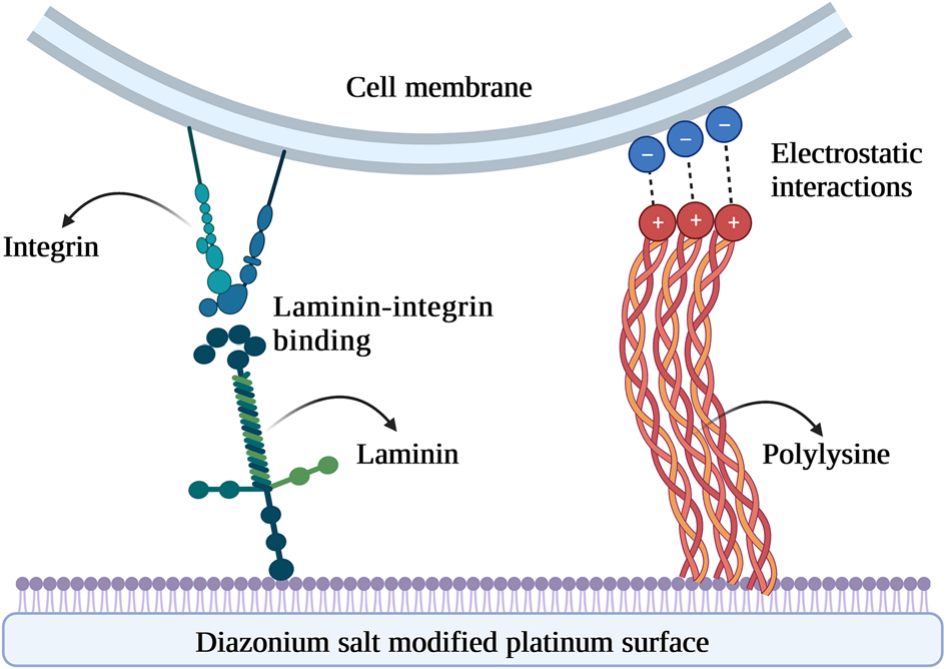
Schematic representation of the interactions between a cell membrane and polypeptides attached to a modified platinum electrode.

The superior pro-adhesive nature of Pt electrodes covalent modified with a mixture of PL and LM arises from different types of interactions of these molecules with a cell membrane (Fig. 6). The interactions of cell membrane with polylysine are physical in nature, and are based on electrostatic effects occurring between negatively charged ions of the cell membrane and positively charged polylysine^46^. When polylysine is immobilized on Pt, it increases the number of positively charged sites available for cell binding. However, laminin functions differently as it directly interacts with a cell membrane through integrins. This type of interactions involves the α integrin subunit ectodomain and the CD151 EC2 domain in a protein-to-protein binding manner^47^. Due to their different molecular targets, the combination of PL and LM chemistry (particularly when attached covalently to the surface) can serve as a potent functionalization route enhancing cell adhesion. The facilitating effect of introduced surface chemistry on cell adhesion is further enhanced by the increase in roughness and wettability, which is observed when compared with unmodified Pt electrode. Due to the complexity of the cell adhesion mechanism, involving both physical and chemical factors, it is not possible to assess the individual role of each of them in the process of cell adhesion.

## Conclusion

This work aims to introduce an electrochemistry-based biofunctionalization technique employing diazonium salts and polypeptides to fabricate a pro-adhesive electrode coating. For this purpose, the surface of a platinum-coated thermanox electrode was pre-modified with the use of a “molecular glue” i.e., electrochemically reduced 4-nitrobenzenediazonium salt further converted into aminobenzene moiety, and coated with PL and PL/LM through either a drop casting method or a covalent attachment. The results of this study confirmed the role of immobilized PL and PL/LM as promotors of cell adhesion. Cell adhesion results emphasized on PL and PL/LM coupled surfaces being superior cell culture platforms, as evidenced by their enhanced roughness and moderate hydrophilicity. Both SH-SY5Y and U87 cells showed increased adhesion on the samples functionalized with covalently bonded polypeptides. Further application of a covalent coupling mechanism can be used to employ a variety of peptides and polypeptides as adhesion promoters. Although extensive research has been done on diazonium salts, the approach introduced by us led to a new pathway of the formation of biofunctionalized electrodes showing enhanced cell adhesion and applicability in therapies based on electrical stimulation.

### Supplementary Information

Supplementary Information.

## Data Availability

Data will be made available on request from a corresponding author, Katarzyna Krukiewicz (katarzyna.krukiewicz@polsl.pl).
