# Supplementary material for: Modulating pro-adhesive nature of metallic surfaces through a polypeptide coupling via diazonium chemistry
Source: Sci Rep. 2023 Oct 26;13:18365. doi: 10.1038/s41598-023-45694-z (PMC10603177; doi:10.1038/s41598-023-45694-z)
Supplement: Supplementary file 1 — Supplementary Information. [file 41598_2023_45694_MOESM1_ESM.pdf]

Modulating pro-adhesive nature of metallic surfaces through a polypeptide coupling via diazonium chemistry: Supporting Information

Taral Patel [1,2], Magdalena Skonieczna [3,4], Roman Turczyn [1,5], Katarzyna Krukiewicz [1,5,*]

[1] Department of Physical Chemistry and Technology of Polymers, Silesian University of Technology, M. Strzody 9, 44-100 Gliwice, Poland
[2] Joint Doctoral School, Silesian University of Technology, Akademicka 2A, 44-100 Gliwice, Poland
[3] Biotechnology Centre, Silesian University of Technology, Krzywoustego 8, 44-100 Gliwice, Poland
[4] Department of Systems Biology and Engineering, Silesian University of Technology, Akademicka 16, 44-100 Gliwice, Poland
[5] Centre for Organic and Nanohybrid Electronics, Silesian University of Technology, Konarskiego 22B, 44-100 Gliwice, Poland

* Correspondence: Katarzyna Krukiewicz, katarzyna.krukiewicz@polsl.pl

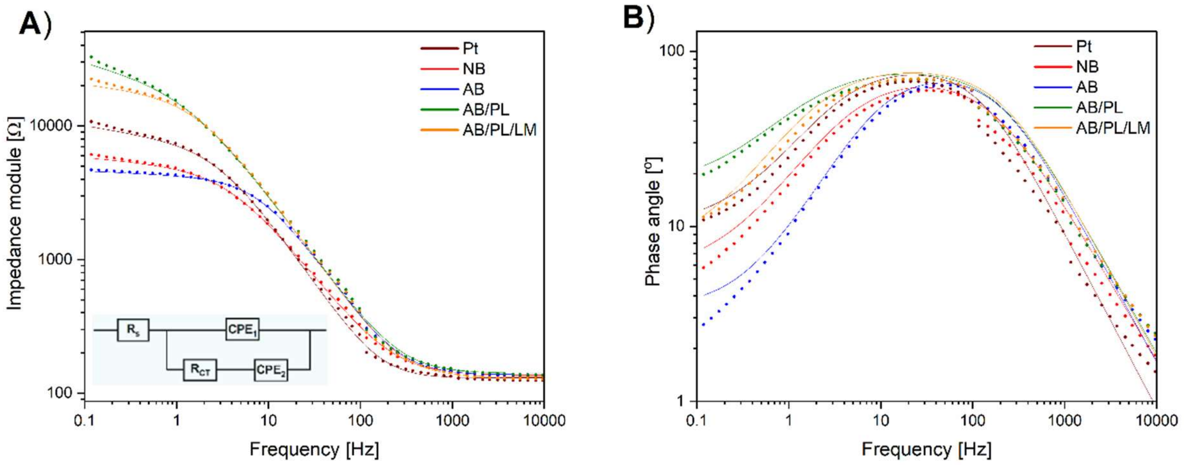

Figure S1. EIS data in the form of Bode plots: impedance modulus vs. frequency (inset: modifie Randles circuit) (A); phase angle vs. frequency (B). Experimental data are represented by dots, results from fitting are represented by lines.

Table S1. Summary of electrical properties of modified layers evaluated from the analysis of EIS data with the use of an equivalent circuit model: R_s – solution resistance, R_{CT} – charge transfer resistance, P_1,n_1 – parameters referring to a constant phase element (CPE_1), P_2,n_2 – parameters referring to a constant phase element (CPE_2).

	R_s	R_{CT}	$P_1 \cdot 10^6$	n_1	$P_2 \cdot 10^4$	n_2	Goodness of fit (%)
**Pt**	130 ± 5	1000 ± 495	7.65 ± 0.44	0.99 ± 0.01	1.17 ± 0.08	0.11 ± 0.03	9.4
**NB**	136 ± 4	3104 ± 212	11.81 ± 0.48	0.89 ± 0.01	3.99 ± 0.44	0.11 ± 0.04	6.4
**AB**	137 ± 3	3345 ± 134	5.91 ± 0.22	0.96 ± 0.01	9.30 ± 1.67	0.13 ± 0.06	5.8
**AB/PL**	141 ± 6	9932 ± 1911	6.68 ± 0.36	0.93 ± 0.01	0.05 ± 0.01	0.23 ± 0.06	10.1
**AB/PL/LM**	132 ± 6	2000 ± 1340	6.14 ± 0.34	0.95 ± 0.01	0.55 ± 0.05	0.07 ± 0.03	10.2
